# Bio-inspired temporal regulation of ion-transport in nanochannels†

**DOI:** 10.1039/c8na00414e

**Published:** 2019-03-12

**Authors:** K. P. Sonu, Sushmitha Vinikumar, Shikha Dhiman, Subi J. George, Muthusamy Eswaramoorthy

**Affiliations:** Nanomaterials and Catalysis Laboratory, Chemistry and Physics of Materials Unit, School of Advanced Materials (SAMat), Jawaharlal Nehru Centre for Advanced Scientific Research Jakkur P.O. Bangalore 560064 India eswar@jncasr.ac.in; Supramolecular Chemistry Laboratory, New Chemistry Unit, School of Advanced Materials (SAMat), Jawaharlal Nehru Centre for Advanced Scientific Research Jakkur P.O. Bangalore 560064 India george@jncasr.ac.in subijg@gmail.com

## Abstract

Temporal regulation of mass transport across the membrane is a vital feature of biological systems. Such regulatory mechanisms rely on complex biochemical reaction networks, often operating far from equilibrium. Herein, we demonstrate biochemical reaction mediated temporal regulation of mass transport in nanochannels of mesoporous silica sphere. The rationally designed nanochannels with pH responsive electrostatic gating are fabricated through a hetero-functionalization approach utilizing propylamine and carboxylic acid moieties. At basic pH, cationic small molecules can diffuse into the nanochannels which release back to the solution at acidic pH. The transient ion transport is temporally controlled using a base as fuel along with esterase enzyme as the mediator. The slow enzymatic hydrolysis of a dormant deactivator (ethyl acetate) determines the lifetime of transient encapsulated state, which can be programmed easily by modulating the enzymatic activity of esterase. This system represents a unique approach to create autonomous artificial cellular models.

## Abstract 2

Mesoporous silica showing pH responsive gating was coupled with an enzymatic reaction to achieve the temporal regulation of ion transport.

## Graphical abstract

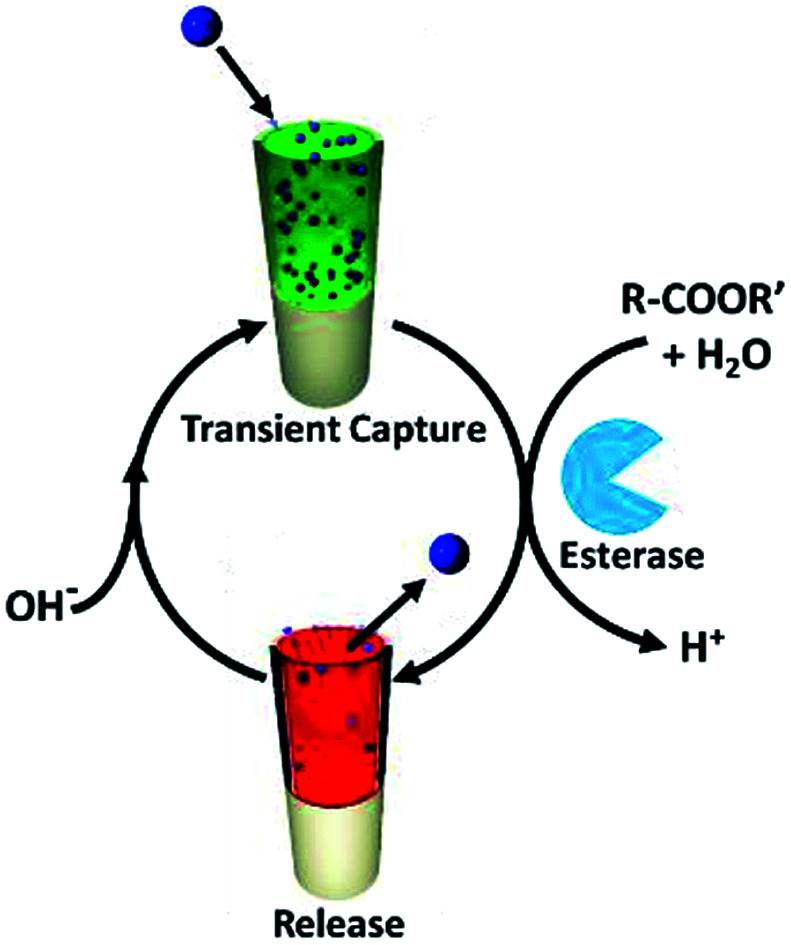

## Introduction

Biological systems have been a long standing inspiration for temporal control over self-assembled nanostructures and functions. From precise control over circadian rhythms to translocation of molecules across membranes, natural systems achieve the same by complex loops of chemical reaction networks facilitated by the cascade of enzymes resulting in structural changes that execute corresponding functions of the cellular motifs.^1,2^ Inspired by this, a recent move towards complex adaptive materials with temporal control over structure and function has begun. Many active self-assembled materials have been synthetically designed recently *via* fuel-driven, non-equilibrium structural/conformational changes.^3–6^ However, autonomous, temporal control on biomimetic functions still remain a challenge and needs to be addressed to design life-like materials.

Recent attempts to achieve temporal regulation are, in general, concerned with transient assembly or conformational changes of supramolecular polymers and of colloidal systems.^7–18^ Most of these systems are examples of fundamental design strategies to build a transient change in self-assembled structures. However, temporally programmed functions similar to natural systems are very few.^19–22^ Prins and co-workers designed systems showing transient signalling and transient catalysis.^23–25^ Using a transient pH switch, Walther and co-workers have exemplified the applications in out-of-equilibrium photonics.^26^ Very recently Ulijn and co-workers elegantly designed enzyme responsive chromophores for transient electronics.^27^

**Scheme 1:**
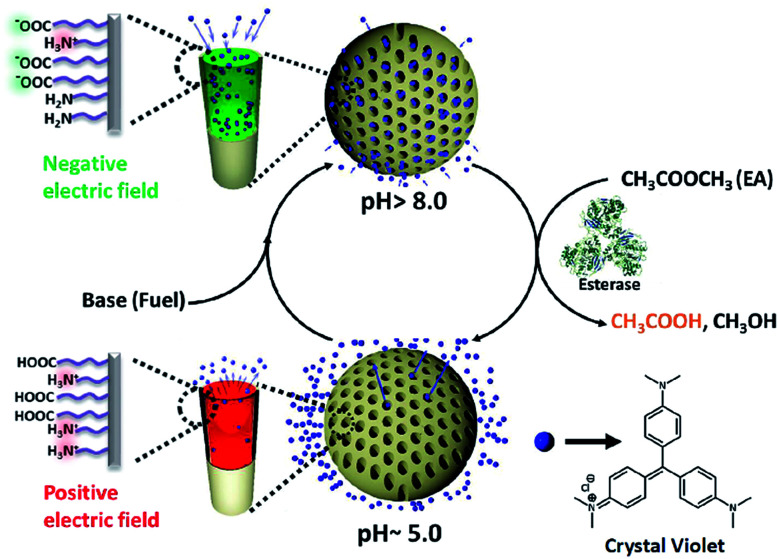
Bio-inspired enzyme regulated temporal regulation of ion transport in nanochannels: the pH responsive charge reversal in mesoporous silica nanochannels (MCM-Z) is integrated with a non-linear pH modulating enzymatic reaction – ethyl acetate (dormant deactivator, DD) hydrolysis by esterase. At the equilibrium state (pH ∼ 5.0), the transport of the cationic dye (crystal violet, CV^+^) is blocked due to electrostatic repulsion. Injection of chemical fuel (base) along with dormant deactivator (ethyl acetate) reverses the surface charge of the compartments, which leads to the uptake of the CV^+^. At the basic pH, the enzymatic hydrolysis (catalysed by esterase present in the system) of ethyl acetate (DD) brings the pH down to acidic pH that subsequently release the dye from the nanochannels. The transient ion transport can be temporally programmed by variation of concentration of the chemical components.

Amongst a plethora of autonomous functions, selective transport of molecules in and out of the cellular compartments is a vital feature of biological systems. In order to achieve this selective transport, cell employs various sophisticated membrane channel proteins.^28^ There have been significant efforts to mimic such selective transport of molecules across the cell membrane in artificial nanochannels.^29–33^ However, all such artificial systems that demonstrated selective transport of molecules often operate in a passive manner and lack autonomous behaviour, unlike their natural counterparts.^34–45^ To alleviate this scenario, herein we report a unique temporal regulation of ion transport in synthetic nanochannels using a bio-inspired, chemical fuel-driven strategy. In order to show this concept of ‘transient ion transport’, we have used pH responsive, heterogeneously functionalized nanochannels of mesoporous silica particles as a model system (Scheme 1). Moreover, we obtain a control over the slow release of encapsulated cationic molecule which can be used for controlled payload/drug release.

## Results and discussion

**Fig. 1:**
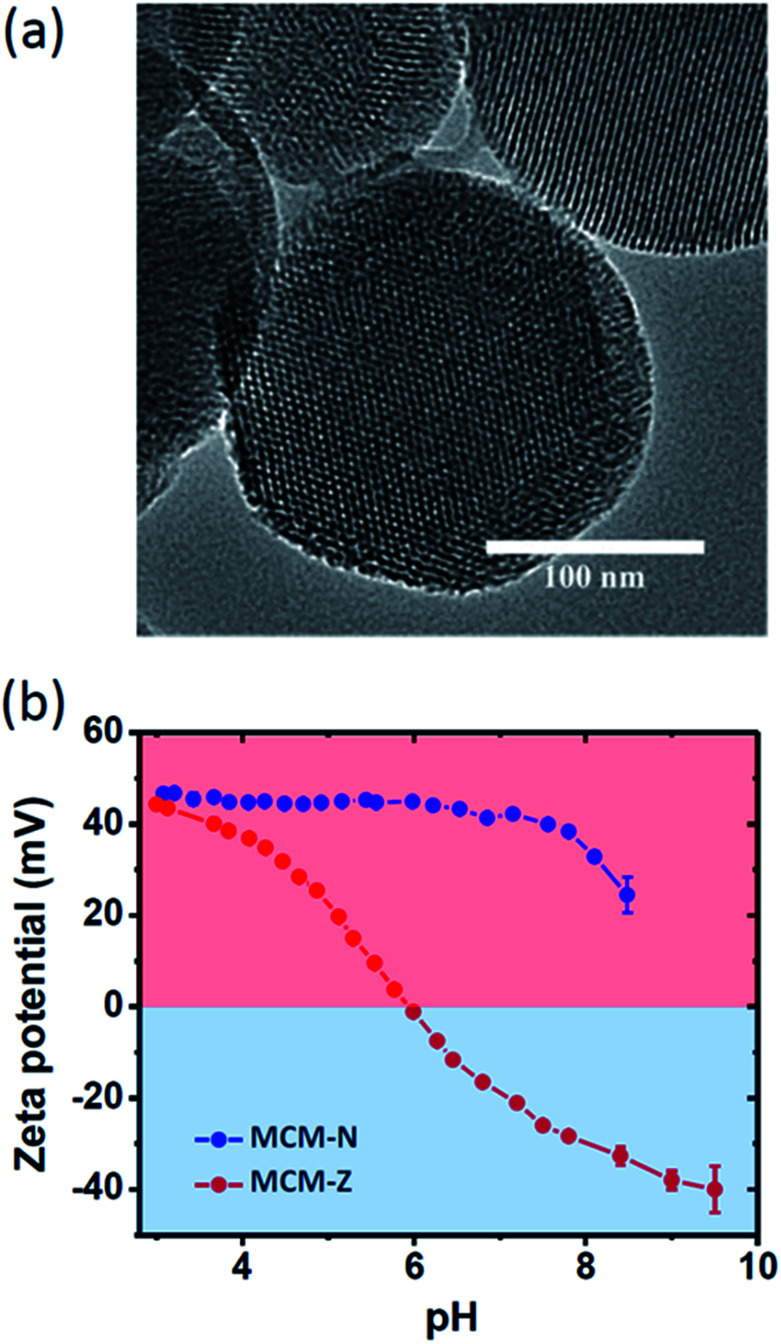
(a) TEM image of MCM-Z. (b) pH dependent zeta potential for MCM-N and MCM-Z.

For a transient ion transport, the first condition is to synthesize charge-switchable nanochannels that can undergo charge reversal on the addition of an external chemical fuel (activation). The second requirement is to have a chemical reagent that can revert the changes in a temporal manner (deactivation). Importantly, the steps should work in tandem, with the rate of activation higher than the rate of deactivation. As the chemical fuel consumed, rate of deactivation becomes higher and the system comes to equilibrium. For this, we have heterogeneously functionalized nanochannels of mesoporous silica particles with pH responsive amine and carboxylic acid functional groups (Scheme 1). Mesoporous silica spheres (MCM-41, hereafter referred as MCM) (Fig. S1 and S2†) were synthesized following a well-known sol–gel procedure and the surface was covalently modified with (3-aminopropyl)triethoxysilane to obtain MCM-N with amine functionalized surface.^46,47^ A portion of amine groups was then reacted with succinic anhydride to form MCM-Z with zwitterionic nature having carboxyl and amine groups on the nanochannel surface (Fig. S1†). The XRD patterns of MCM, MCM-N and MCM-Z exhibited a small angle peak (at around 2*θ* = 2.5 degree) suggesting that the hexagonal mesostructure was retained during functionalization (Fig. S3†). Retention of mesoporosity of nanochannels was further confirmed *via* transmission electron microscopy (Fig. 1a). Thermogravimetric analysis (TGA) suggested propylamine functionalization in MCM-N was around *ca.* 2.0 mmol g^−1^ (Fig. S4†). The carboxylic acid groups (–COOH) in MCM-Z was determined to be around *ca.* 1.1 mmol g^−1^ using TGA and ninhydrin test (Fig. S5†), which suggest that approximately 56% of amine groups were covalently connected (*via* amide linkage) to carboxylic acid groups in MCM-Z.

Nitrogen adsorption–desorption analyses were carried out at 77 K to evaluate the progress of functionalization inside the nanochannels (Fig. S6a†). The Barrett–Joyner–Halenda (BJH) pore size distributions showed a gradual reduction in average pore size from *ca.* 2.9 nm for MCM to 2.5 nm for MCM-N which further decreased to 2.2 nm for MCM-Z suggesting progress of functionalization inside the nanochannels (Fig. S6b†). Further, the number of propylamine groups on MCM-N was calculated to be 1.25 molecules per nm^2^ area from N_2_ sorption studies and TGA (see ESI† text for details). Similarly, in the case of MCM-Z, the number of carboxylic acid groups present was around 0.71 molecule per nm^2^.

To monitor the surface charge of these biomimetic nanochannels, we employed zeta potential measurements. The variation of zeta potential with pH for both MCM-N and MCM-Z were shown in Fig. 1b. As expected, propylamine functionalized MCM (MCM-N) showed a zeta potential of *ca.* +46 ± 0.5 mV at pH 3 that reduced to +24 ± 2 mV at pH 8.5. In the case of MCM-N, no charge reversal occurred on going from low pH to high pH (Fig. 1b, S7†). On the other hand MCM-Z (that have both amine groups and carboxylic acid groups on the surface) showed a clear charge reversal with respect to pH. At pH 3, MCM-Z was positively charged with a zeta potential of *ca.* +44 ± 0.5 mV associated with the protonation of free amines in MCM-Z. The surface charge became zero at around pH 6 (isoelectric point) and a further increase in pH caused charge reversal from positive to negative with a zeta potential of *ca.* −32 ± 1 mV at around pH 8.5. This can be attributed to the generation of carboxylate groups along with the neutralization of amines. As a result, the functionalized nanochannels become anionic above the isoelectric point of pH 6.0 and cationic below pH 6.0.

**Fig. 2:**
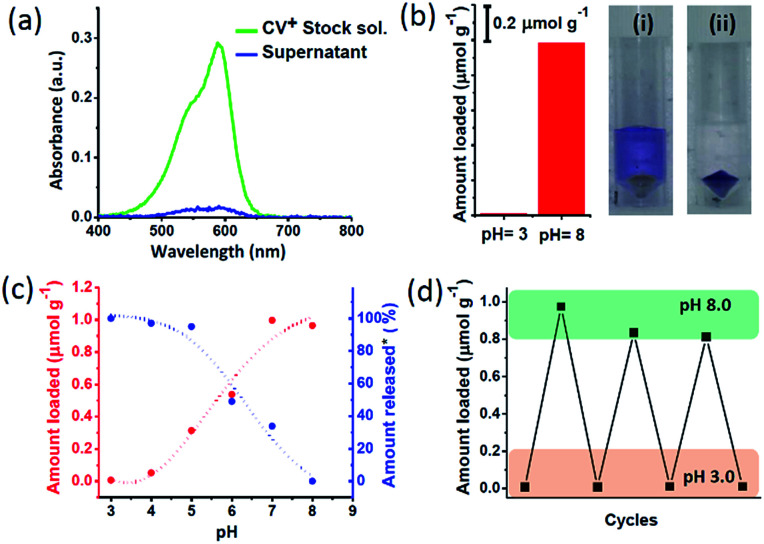
Transportation of CV^+^ in MCM-Z nanochannels: (a) absorption spectra of CV^+^ stock solution and the supernatant after incubating with MCM-Z at pH 8. (b) Amount of encapsulation of CV^+^ into MCM-Z at pH 3 and pH 8 (CV^+^ stock solution concentration was *ca.* 3.34 μM and MCM-Z was *ca.* 10 mg). Photographs showing the supernatant solution after soaking CV^+^ with MCM-Z for 10 min in (i) pH 3 and (ii) pH 8. (c) Encapsulation and release of CV^+^ in MCM-Z at different pH. *For release studies, CV^+^ was first encapsulated in to MCM-Z at pH 8 and the released amount at different pH was normalized w.r.t. loading at pH 8. (d) Cycling of CV^+^ encapsulation into MCM-Z at pH 3 and 8 (see ESI† for the details).

The pH dependent charge switchable property of nanochannels was explored to encapsulate and release of cationic dye molecule. For this purpose, a cationic dye – crystal violet (CV^+^) was chosen which maintain the cationic nature in the pH range of 3 to 8.5.^48,49^ The dye uptake and release were quantitatively probed *via* UV-Vis spectroscopy (Scheme 1, Fig. S8†). To investigate the amount of CV^+^ encapsulation, 10 mg MCM-Z was soaked in a 3.34 μM aqueous solution of CV^+^ dye at pH 8 for 10 minutes. The absorption spectra of CV^+^ stock solution and supernatant solution after soaking with MCM-Z was compared (Fig. 2a). The disappearance of CV^+^ absorption band for supernatant solution suggested an efficient encapsulation of cationic dye in negatively charged nanochannels at pH 8 *via* electrostatic attraction. The encapsulation is driven by electrostatic attraction between the CV^+^ and silica surface. There are no physically entrapped dye molecules inside the pore as it is evident from ‘zero’ leak release of CV^+^ from silica pores at pH > 8.0 (Fig. 2c). The uptake of CV^+^ calculated from the UV-vis spectra was found to be *ca.* 1 μmol g^−1^ (Fig. 2b). Similar encapsulation experiment was done at pH 3 and only a negligible uptake was observed, confirming the strong electrostatic gating of MCM-Z nanochannels at pH 3 towards the cationic dye (Fig. 2b).

Next we studied the CV^+^ dye uptake in MCM-Z and its release as a function of pH (Fig. 2c). The MCM-Z (10 mg) was incubated in a 3.34 μM CV^+^ solution at different pHs (in the range of 3–8) for 10 minutes and the encapsulation was probed with absorption spectra. A gradual increase in the uptake of CV^+^ was observed with increasing pH and a maximum uptake was seen at pH 8 (*ca.* 1 μmol g^−1^) which can be explained based on the nature of surface charge developed at different pH as shown in Fig. 1b. Release profile of CV^+^ encapsulated in MCM-Z at pH 8.0 (high uptake due to electrostatic interactions) showed maximum release at pH 3.0 due to the charge repulsion between the positively charged nanochannels and the cationic dye (Fig. 2c). We further investigated the recyclability of the charge reversal and dye encapsulation by sequentially changing the pH of the MCM-Z dispersion containing 1 μmol g^−1^ CV^+^ between 3 and 8. The encapsulation and release of CV^+^ in and out of MCM-Z was studied and a significant amount of dye uptake was observed up to three cycles (>80%) (Fig. 2d). Thus, we obtained an efficient recycling, validating the adaptability and reversibility of these nanochannels to solution pH variations.

Further, we investigated the structural stability of MCM-Z in basic aqueous solution (pH > 8.0) to evaluate the robustness of the nanochannels. The MCM-Z was dispersed in Tris buffer of pH 9.0 (10 mM) and the zeta potential was measured at regular time intervals up to 3 hours, which showed no change even after 2.5 hours (Fig. S9a†). The XRD pattern (Fig. S9b†) and N_2_ sorption analysis (Fig. S9c†) also confirmed the structural stability of MCM-Z nanochannels in basic conditions.

**Fig. 3:**
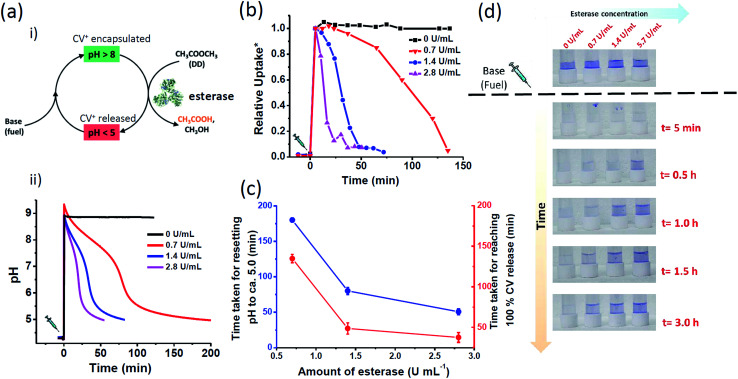
Enzyme regulated temporal control of CV^+^ encapsulation in MCM-Z: (a) (i) schematic showing transient ion transport with chemical fuel-driven activation and ethyl acetate (DD) hydrolysis by esterase as deactivation step. (ii) The temporal change in pH of the system containing MCM-Z, CV^+^ and esterase. The equilibrium state pH is *ca.* 4.2 and addition of chemical fuel base (Tris, 17 mM final concentration) along with ethyl acetate (DD, 66 mM final concentration) increases the pH of the system to *ca.* 9.0. The transient basic pH state slowly comes back to equilibrium state *ca.* 5.0 as esterase catalyzed hydrolysis of ethyl acetate proceeds. (b) Temporal regulation of CV^+^ uptake in MCM-Z. The lifetime of CV^+^ encapsulated state is programmed by concentration of esterase. (c) Comparison between the lead time (*t*_lead_, time taken for resetting pH to *ca.* 5.0) and response time (*t*_response_, time taken for reaching 100% CV^+^ release). (d) Photographs showing enzyme regulated temporal variation of the colour of supernatants. * The CV^+^ encapsulation is normalized w.r.t. amount of encapsulation at [esterase] = 0 U mL^−1^.

With the understanding of switchable charged nanochannels of MCM-Z at varying pH between 3 to 8 and corresponding cationic dye encapsulation and release, we next envisioned to couple this to a temporal change in pH to realize “transient ion transport”. For this, we have exploited a feedback-controlled chemical processes which temporally modulates the pH of the surrounding solution.^50^ When we first inject a base to the acidic dispersion of MCM-Z, the increase in pH (∼9.0) deprotonates the carboxylic acid functional groups (and ammonium cations) resulting in the formation of anionic nanochannels (activation). Furthermore, the high pH initiates hydrolysis of ethyl acetate (by esterase enzyme) that gradually decreases the pH (due to the formation of acetic acid). As a result amine and carboxylate functional groups get protonated and nanochannels become cationic (deactivation). Since ethyl acetate decreases the pH of the system only *via* its kinetically controlled hydrolysis (by esterase enzyme) it is referred as dormant deactivator. The instantaneous activation (formation of anionic nanochannels) by the addition of base and the slow deactivation (leading to cationic nanochannels) due to ethyl acetate hydrolysis allow the nanochannels to exist in transient anionic state (Fig. 3a(i)).

We first investigated the efficiency to obtain temporal control on pH by varying the amount of esterase. The chemical fuel (tris(hydroxymethyl)aminomethane (base)) and ethyl acetate (DD) in appropriate concentrations were mixed and injected to a dispersion of MCM-Z containing ∼1.0 μmol g^−1^ CV^+^ and esterase enzyme (0–3 U mL^−1^) and a time dependent pH variation was monitored (Fig. 3a(ii)). An initial increase in pH to ∼9 was observed that gradually decayed to equilibrium state acidic pH ∼5.0 due to the formation of acetic acid by the hydrolysis of ethyl acetate (DD). The kinetics of ethyl acetate hydrolysis is exclusively proportional to the concentration of esterase. The increase in concentration of enzyme (at constant concentration of ethyl acetate) resulted in higher rate of ethyl acetate hydrolysis and a corresponding decrease in lifetime of transient alkaline pH state. For *ca.* 0.7 U mL^−1^ of esterase enzyme, it took more than 180 ± 3 min to return the pH back to *ca.* 5; however, it took only 50 ± 4 min when the enzyme amount was increased to *ca.* 2.8 U mL^−1^ (Fig. 3c). On the other hand, the pH remained around 9.0 when no esterase was used attributing the importance of esterase enzyme to build transient profile. Interestingly, the temporal variation of solution pH showed three distinct slopes which were in agreement with the previously reported activity profile of esterase.^50^

The temporal control achieved on pH was next coupled to CV^+^ dye encapsulation in MCM-Z. Due to the above described *in situ* modulation of pH, the permeation of ions in and out of the pH adaptive MCM-Z nanochannels was expected to adjust autonomously (Fig. 2b, d). To achieve a temporal control on ion transport, MCM-Z, CV^+^ and esterase enzyme at pH 4.2 were taken. The system stays at equilibrium and insignificant encapsulation of CV^+^ by MCM-Z was observed even after 10 min of soaking (Fig. 3b). However, on addition of chemical fuel base (Tris) and ethyl acetate, resulted in an instantaneous increase in pH that facilitated a spontaneous CV^+^ encapsulation in MCM-Z nanochannels. Subsequently, hydrolysis of ethyl acetate (DD) by esterase occurs that gradually decreased the pH and a slow CV^+^ release back into the solution is observed. Thus, a transient ion transport or transient molecule encapsulation is attained.

Further, the lifetime of the transient ‘CV^+^ encapsulated state’ was programmed by modulating deactivation rate *via* enzyme concentration. In the case of 2.8 U mL^−1^ esterase concentration, within 38 ± 6 min complete release was seen; on the other hand, when the esterase concentration was reduced to 0.7 U mL^−1^, it took longer time to reach complete release (>135 ± 5 min) (Fig. 3b–d). This clearly demonstrates the temporal programmability of CV^+^ encapsulation and modular transient ion transport. Interestingly, we noted that for any esterase concentration, time taken for resetting pH back to the equilibrium pH (*i.e.* 5.0) (*t*_lead_) was higher than the time required for the complete release (*t*_response_) (Fig. 3c).

**Fig. 4:**
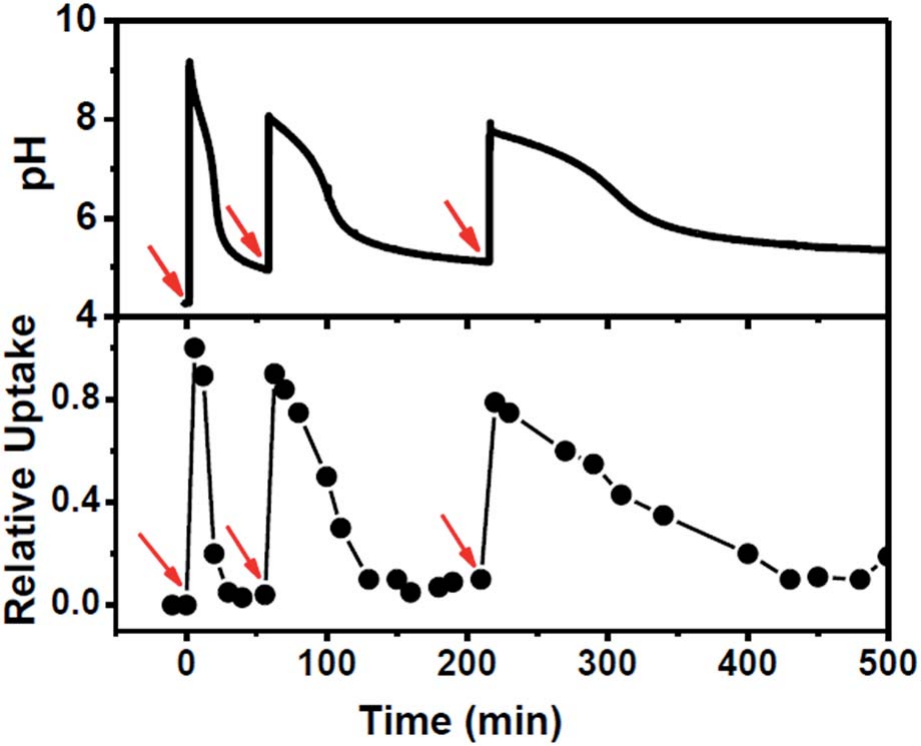
Refuelling the system showing the recyclable nature of CV^+^ encapsulation and release in MCM-Z along with corresponding pH trace. Red arrows indicate addition of fuel along with ethyl acetate (DD). Addition of base (fuel) increased pH of the dispersion and at basic pH ethyl acetate hydrolysis mediated by esterase decrease the pH gradually.

The system was further scrutinized to test its ability to undergo multiple cycles on refuelling by chemical fuel (Tris) (Fig. 4). For this purpose, a dispersion containing MCM-Z, CV^+^ (∼1.0 μmol g^−1^) and 2.8 U mL^−1^ esterase was taken at a pH around 4.2 and the pH and CV^+^ uptake was monitored (Fig. 4). As expected no uptake was observed at this equilibrium state. However, an instantaneous encapsulation of CV^+^ was observed up on the addition of chemical fuel (Tris) (same amount used in the previous experiments). The CV^+^ uptake was followed by gradual release as ethyl acetate (DD) hydrolysis proceeds (ethyl acetate was added along with chemical fuel). Once the system reached back to the equilibrium state, it was refuelled by adding same amount of chemical fuel (Tris) and ethyl acetate (DD) and a similar temporal profile was observed. Third refuelling also resulted in a similar trend. Thus system could be refuelled multiple times by subsequent addition of fuel, however, there was a dampening observed in the time required for complete release of CV^+^ as the cycles proceeded. This can be attributed to the accumulation of waste products in the closed system. Nevertheless, we could obtain a transient ion transport and successfully modulate its temporal profile and refuel it multiple times.

## Conclusions

In summary, we demonstrated the construction of mesoporous silica based biomimetic nanochannels having enzyme mediated autonomous (temporal) regulation over ion transport. The fuel driven encapsulation of ions into the nanochannels and temporal control of release have been shown for the first time in such nanochannels. A modular lifetime of transient ion transport was shown along with the refuelability of the system. The nanochannels were also found to be very robust in terms of structural stability in basic medium. These temporally switchable and adaptive nanochannels shall pave the way for future on demand devices or delivery vehicles.

## Conflicts of interest

There are no conflicts to declare.

## Supplementary Material

NA-001-C8NA00414E-s001
