# Supplementary material for: Bio-inspired temporal regulation of ion-transport in nanochannels
Source: Nanoscale Adv. 2019 Mar 12;1(5):1847–52. doi: 10.1039/c8na00414e (PMC9418411; doi:10.1039/c8na00414e)
Supplement: NA-001-C8NA00414E-s001 [file NA-001-C8NA00414E-s001.pdf]

Electronic Supplementary Material (ESI) for Nanoscale Advances.
This journal is © The Royal Society of Chemistry 2019

Supporting Information

Bio-inspired Temporal Regulation of Ion-Transport in Nanochannels

K. P. Sonu,† Sushmitha Vinikumar,† Shikha Dhiman,‡ Subi J. George,‡, * and Muthusamy Eswaramoorthy†, *

(†) Nanomaterials and Catalysis Laboratory, Chemistry and Physics of Materials Unit and School of Advanced Materials (SAMat)

(‡) Supramolecular Chemistry Laboratory, New Chemistry Unit and School of Advanced Materials (SAMat), Jawaharlal Nehru Centre for Advanced Scientific Research, Jakkur P.O., Bangalore 560064, India

Table of Contents

General characterization and equipment

Materials

Methods

Supporting Figures (S1-S9)

References

General characterization and equipment

Powder XRD patterns were recorded using Bruker-D8 diffractometer using Cu Kα radiation, (=1.54 Å, Step size: 0.02, Current: 30 mA and Voltage: 40 kV). The N_2 adsorption studies at 77 K were performed on a Autosorb-1C (Quantachrome corp.). The samples were outgassed at 90 °C for 12 h under high vaccum before the analysis. Ultrahigh purity gases (99.9995%) were used for all experiments. Thermogravimetric analysis experiments were performed using Mettler Toledo 850 from 30 °C to 1000 °C in oxygen stream with a heating rate of 10 °C/min. FT-IR spectra were recorded on a Bruker IFS 66v/S spectrometer. FE-SEM images were obtained by means of Nova-Nano SEM-600 (FEI, Netherlands). Electronic absorption spectra were recorded on a Perkin Elmer Lambda 900 UV-Vis-NIR spectrometer. TEM images were recorded on a JEOL JEM- 3010 electron microscope with an applied acceleration voltage of 300 kV. Zeta potential measurements were carried out using a Nano ZS (Malvern UK) employing a 532 nm laser at a back scattering angle of 173°. pH measurments were recorded on a Thermo Scientific pH Meter STAR A211.

Materials

N- Cetyltrimethylammonium bromide (CTAB), tetraethyl orthosilicate (TEOS, 99%), (3-aminopropyl)triethoxysilane (APTES, 99%), crystal violet (CV), tris(hydroxymethyl)aminomethane, esterase from porcine liver, citric acid and trisodium citrate were purchased from Sigma-Aldrich and used as received. Sodium hydroxide (NaOH), hydrogen chloride (HCl), succinic anhydride were purchased from SDFCL. Lithium hydroxide was purchased from Merck. Hydrindantin, ninhydrin, ethyl acetate and all solvents were purchased from Spectrochem.

Methods

Synthesis of MCM-41.

Mesoporous silica nanoparticles (MCM-41) were synthesized by following a previously reported procedure[1]. In a typical synthesis, 1.0 g (2.74 mmol) of hexadecyltrimethyl-

ammonium bromide (CTAB) and 0.28 g of NaOH was dissolved in 480 mL of water under stirring and the temperature was raised to 80°C. 5 ml (22.4 mmol) of tetraethyl orthosilicate (TEOS) was added drop wise and the reaction mixture was stirred for 2 h at 80 °C. The white solid product was centrifuged, washed thoroughly with water followed by ethanol, and dried in oven overnight. The surfactant template (CTAB) was removed from the obtained product by refluxing for 6 h in ethanol solution of pH 1. The precipitate was thoroughly washed with water and ethanol.

Synthesis of MCM-N.

A 1.0 g of surfactant free MCM-41 (1.0 g) and 1.0 ml (5.67 mmol) of (3-aminopropyl)triethoxysilane (APTES) were added to 80 ml of anhydrous toluene and ultrasonicated to get a homogenous dispersion. The mixture was refluxed with stirring under N_2 atmosphere for 24 h. The reaction mixture was centrifuged and the precipitate was washed thoroughly with toluene, hexane followed by ethanol, finally dried under high vacuum at 353 K for 6 h.

Synthesis of MCM-Z.

A 0.5 g of MCM-N was dispersed in 25 mL 1,4-dioxane and ultrasonicated for 5 min. To this dispersion, a solution of 60 mg of succinic anhydride (0.6 mmol) in 12.5 mL of 1,4-dioxane was added under stirring. This mixture was heated at 80 ^{o}C for 1 h. The resultant product was centrifuged, and washed thoroughly with water followed by ethanol, and dried in oven overnight.

Preparation of samples for Nitrogen adsorption-desorption.

The samples were degassed at 90 ^{o}C for 12 h under high vacuum and then N_2 adsorption-desorption experiments were carried out at 77 K.

Calculation of carboxylic acid groups in MCM-Z.

The weight of overall organic functionalization in MCM-N was around 11.7 %

Molecular weight of the propylamine group = 58 g/mol.

Amount of amine groups in MCM-N (calculated from TGA) = **2.0 mmol/g.**

The ninhydrin test furnished the content of amine groups in **MCM-Z** around 0.86 mmol/g.

The weight of overall organic functionalization in **MCM-Z** was around 23.1 %.

Molecular weight of succinic acid conjugated propylamine = 159.18 g/mol.

In 1 g of MCM-Z, total amount of organic functionalization = 0.231 g.

Hence, $(0.86 \times 10^{-3} \times 58) + 159.18 \times (x) = 0.231$g

So, x= **1.13 mmol/g.**

So, percentage of carboxylic acid functionalization in MCM-Z = 1.13 / 2.0 = 56.5 %

Ninhydrin test for the estimation of amine groups[2].

Ninhydrin solution was prepared by dissolving 0.266 g (1.49 mmol) of ninhydrin in 10 mL dimethyl sulfoxide (DMSO) followed by the addition of 0.04 g hydrindantin. The mixture was sonicated for 10 min to form clear solution. To this clear solution 3.3 mL of 4 N LiOH/Acetic acid buffer (pH 5). A 1 mL of ninhydrin solution was mixed with different n-propylamine standard solutions (each 1 mL), 5 mg MCM-N dispersed in 1 mL water and 5 mg **MCM-Z** dispersed in 1 mL water. All these solutions are placed in closed containers and heated at 80 ^{o}C for 30 min. After 30 min the reaction mixture was immediately placed in the ice-bath. The mixture was diluted by adding 20 mL of 50 % ethanol, which further diluted three times before doing absorption analysis. The absorbance at 572 nm was used for all calculations.

Calculation of Quantitative Coverage of Functionalization.

For MCM-N:

Amount of propylamine groups present in MCM-N = 2.0 mmol/g (From TGA measurements).

Number of molecules of propylamine = 2.0 mmol x N_A /g

= 1.204 x 10^{21} molecules/g

Surface area of MCM-41 = 965 m^2/g

= 965 x 10^{18} nm^2/g

Number of propylamine groups per nm^2 = 1.204 x 10^{21} molecules / 965 x 10^{18} nm^2

= **1.25 molecules / nm^2**

For **MCM-Z**:

Amount of carboxylic acid groups present in MCM-Z = 1.13 mmol/g

Number of molecules of carboxylic acid moieties = 1.13 mmol x N_A /g

= 6.804 x 10^{20} molecules/g

Number of carboxylic acid groups per nm^2 = 6.804 x 10^{20} molecules / 965 x 10^{18} nm^2

= **0.71 molecules/ nm^2**

Zeta Potential Measurements.

A dispersion of MCM-N or **MCM-Z** with a concentration of 0.5 mg/mL in respective buffers was used for the zeta potential measurements. The zeta titration was carried out by an Autotitrator (MPT 2 Titrator, Malvern Instruments). pH was adjusted using 10 mM stock solutions of HCl or NaOH. The temperature was always kept at 25 ^oC with the help of inbuilt thermostat in Zetasizer Nano ZS.

Passive CV^+ transport studies.

Transport of cationic dye (CV^+) into the channels of **MCM-Z** at different pH buffers are studied as follows. A 10 mg (accurately weighed) of **MCM-Z** was dispersed in 3 mL buffer (15

mM) of respective pH containing approximately 3.34 µM of CV^+. The mixture was agitated in a vortex mixer for 10 min at room temperature. The dispersion was then centrifuged and the supernatants were collected and subjected to absorption spectroscopy to determine the amount of CV^+ loaded to the **MCM-Z** using Beer-Lambert law. The release of entrapped CV^+ from **MCM-Z** was studied similarly. The CV^+ was loaded into **MCM-Z** (10 mg) at pH 8 as discussed before. The mixture was centrifuged and the precipitate was collected. The precipitate was then re-dispersed in pH buffer (15 mM) of respective pH.

CV^+ uptake cycling. The recyclability of **MCM-Z** towards the CV^+ transport was studied as follows. A 10 mg (accurately weighed) of **MCM-Z** was dispersed in 3 mL buffer (15 mM) of pH 3.0 containing approximately 3.34 µM of CV^+. The mixture was agitated in a vortex mixer for 10 min at room temperature. The dispersion was then centrifuged and the supernatants were collected and subjected to absorption spectroscopy to determine the amount of CV^+ loaded. The precipitate was collected and re-dispersed in a buffer (15 mM) of pH 8.0 containing 3.34 µM of CV^+. Again the supernatant was collected for analysis. Before the second cycle, the precipitate was washed in pH 3.0 buffer (15 mM) to remove the loaded CV^+ dye molecules. The precipitate was further subjected to second cycle of CV^+ loading using fresh buffer containing 3.34 µM CV^+ as above stated. Similar washing procedure was repeated for third cycle also.

Temporal regulation of CV^+ transport.

The mixture containing 10 mg of **MCM-Z**, varying amount of esterase enzyme (0 – 3.0 U), 0.3 mM sodium citrate/citric acid buffer (pH 4.2) and 3.34 µM CV^+ (total volume was made to 2.5 mL with water) was taken. The mixture was agitated in a vortex mixer at room temperature. Subsequently, at the predetermined times the mixture was centrifuged and the supernatant was subjected absorption spectroscopy to determine the amount of CV^+ entrapped to **MCM-**

Z. A 0.5 mL of tris buffer containing ethyl acetate (final concentration, 66 mM) was added to the above mixture to activate the system to "ion entrapped state".

Refueling the system.

To demonstrate the refueling capabilities of the system, a mixture containing 10 mg of **MCM-Z**, 2.8 U/mL esterase enzyme, 0.3 mM sodium citrate/citric acid buffer (pH 4.2) and 3.34 µM CV^+ (total volume was made to 2.5 mL with water) was taken. The mixture was agitated in a vortex mixer at room temperature. Subsequently, at the predetermined times the mixture was centrifuged and the supernatant was subjected UV-Vis absorption spectroscopy to determine the amount of CV^+ entrapped to **MCM-Z.** A 0.5 mL of tris buffer containing ethyl acetate (final concentration, 66 mM) was added to the above mixture to activate the system to "ion entrapped state". Once the equilibrium state is reached, another 0.5 mL of tris buffer containing ethyl acetate (final concentration, 66 mM) was added to refuel the system and to activate it to the "ion entrapped state".

Supporting Figures

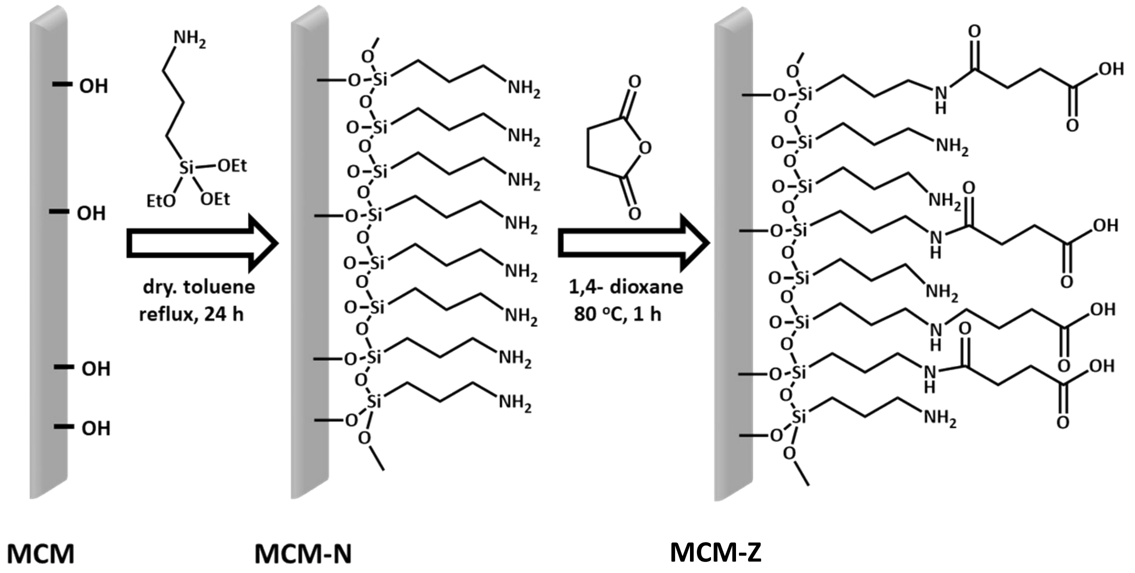

Figure. S1. Schematic showing the synthetic strategy for functionalization of MCM nanochannels to create pH responsive hetero functionalized **MCM-Z** nanochannels.

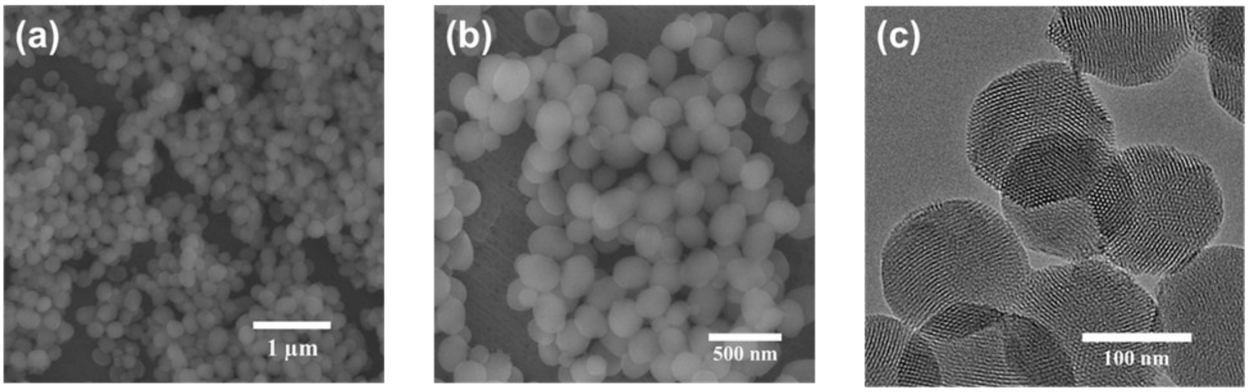

Figure. S2. FE-SEM images of (a-b) MCM at different magnifications. TEM image of (c) MCM.

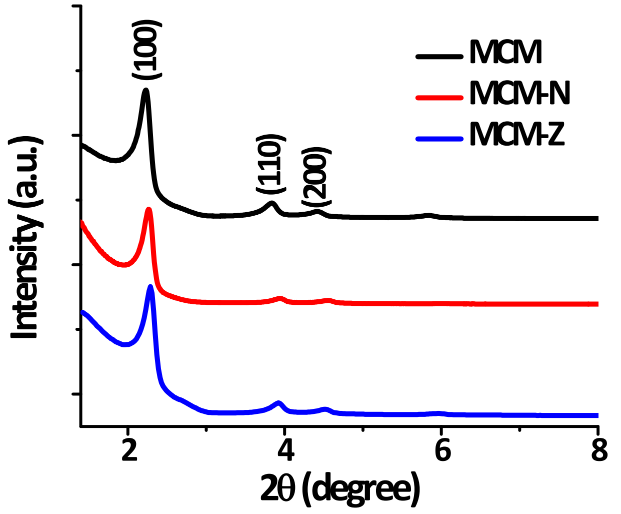

Figure. S3. Powder X-ray diffraction patterns of MCM, MCM-N and **MCM-Z** showing retention of mesostructural ordering with the progress of functionalization.

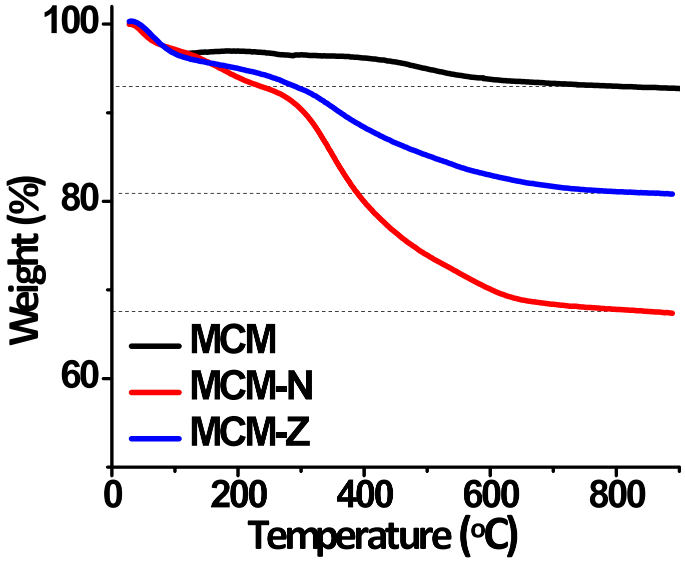

Figure. S4. Thermogravimetric analysis (TGA) curves of MCM, MCM-N and **MCM-Z**.

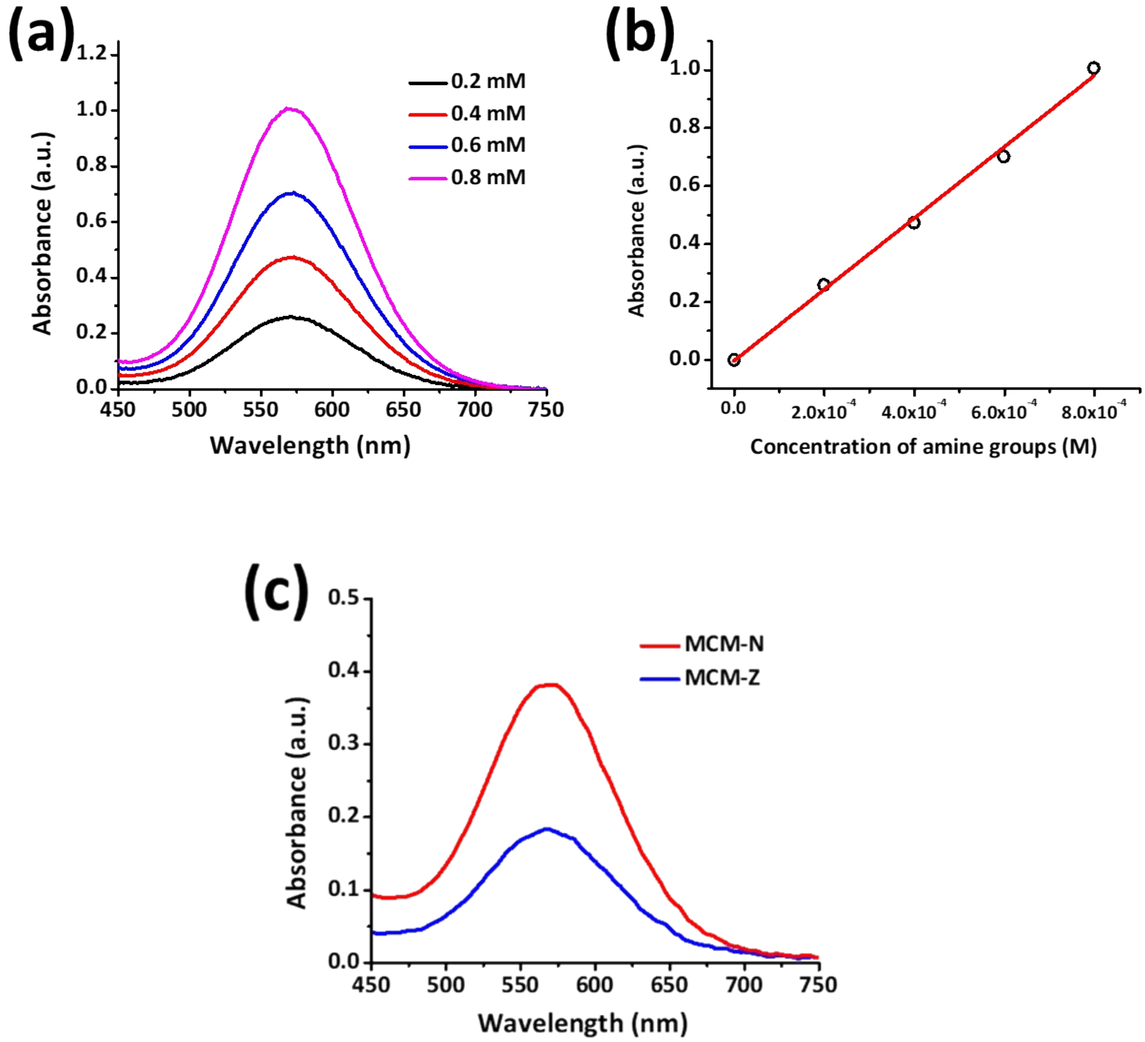

Figure. S5. (a) Absorption spectra of the ninhydrin complex in the propylamine solutions used for calibration. (b) Linear calibration plot showing increase in absorbance with increase in propylamine used in the standard solution. This calibration plot was used to determine the concentration of amine groups in MCM-N and **MCM-Z**. (c) Absorption spectra for the ninhydrin test to quantify the amount of amine groups in MCM-N and **MCM-Z**. Amine functionalization in **MCM-Z** was about half of that in MCM-N.

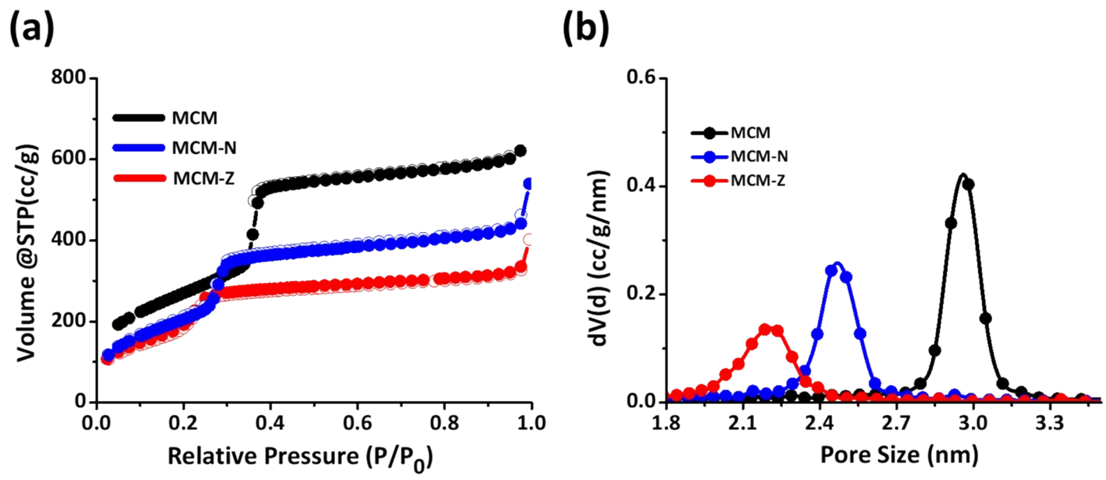

Figure. S6. (a) Nitrogen adsorption-desorption isotherms and (b) Barrett-Joyner-Halenda (BJH) pore size distribution curves of MCM, MCM-N and **MCM-Z**.

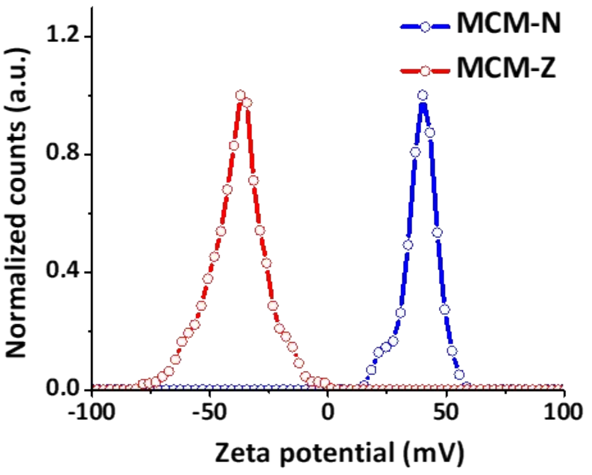

Figure. S7. Normalized zeta potential distribution curves of MCM-N and **MCM-Z** (in 10 mM tris buffer, pH 8.0).

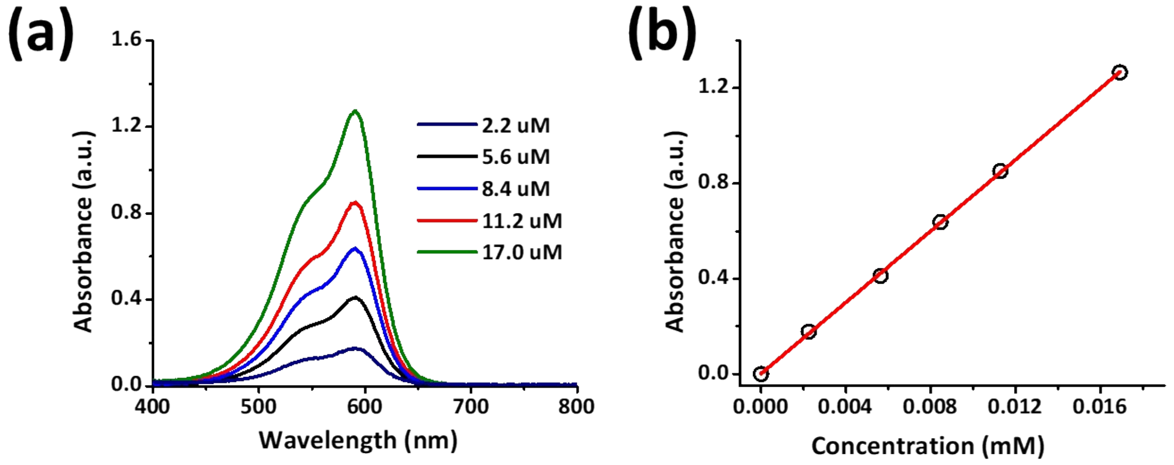

Figure. S8: (a) Concentration dependent absorption spectra of CV^+. (b) Calibration curve used for estimating concentration of CV^+.

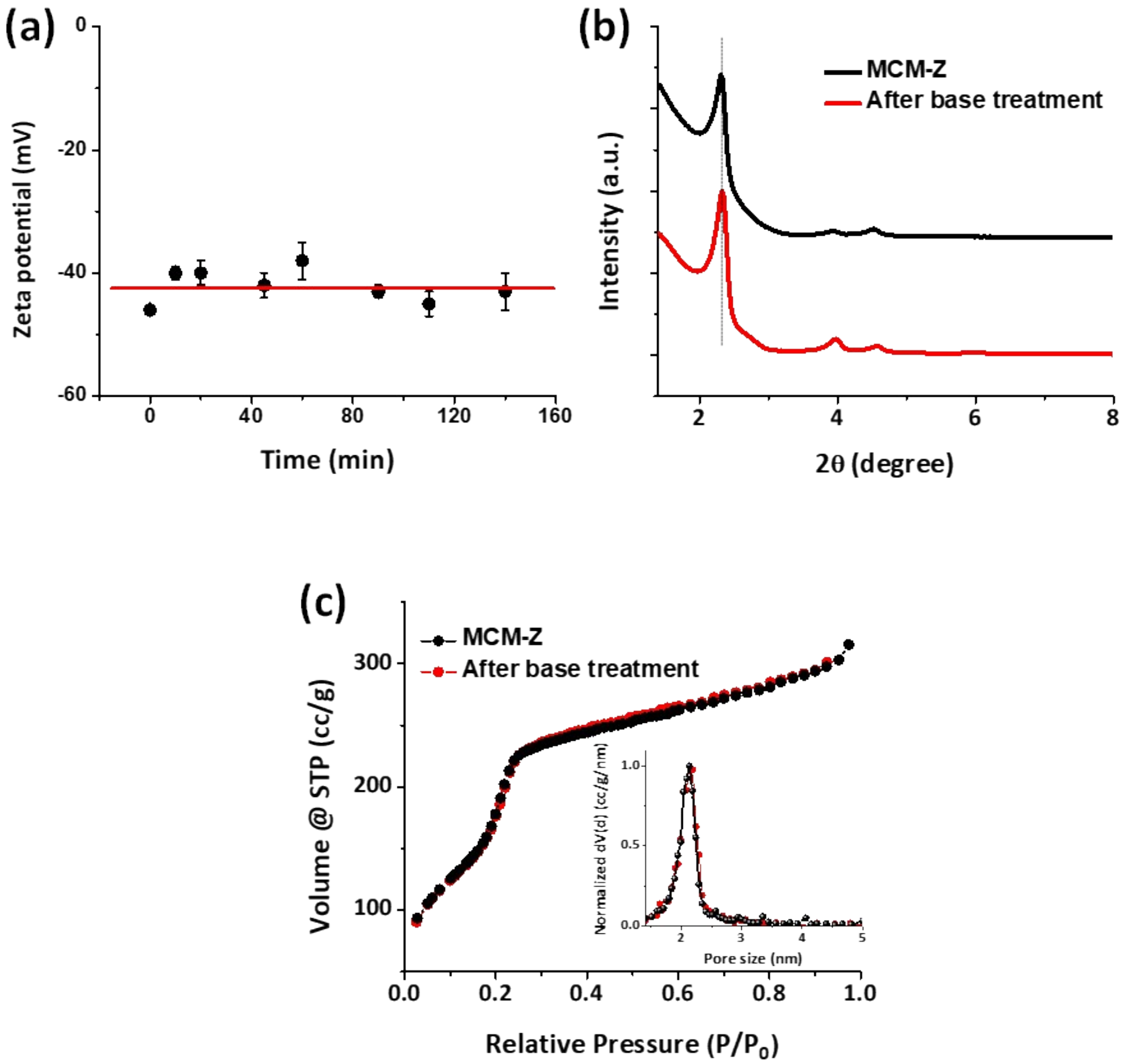

Figure. S9: (a) Zeta potential measurement of **MCM-Z** dispersed in pH 9.0 buffer at different soaking time. (b) XRD pattern of **MCM-Z** before and after soaking in pH 9.0 buffer for 2.5 h. (c) Nitrogen adsorption-desorption isotherms and (inset) Barrett-Joyner-Halenda (BJH) pore size distribution curves of **MCM-Z** before and after soaking in pH 9.0 buffer for 2.5 h.

REFERENCES

[1] S. Huh, J. W. Wiench, J. C. Yoo, M. Pruski, V. S. Y. Lin, *Chem. Mater.* 2003, **15,** 4247-4256.

[2] S. W. Sun, Y. C. Lin, Y. M. Weng, M. J. Chen, *J. Food Comp. Anal.* 2006, **19,** 112-117.
